# The *Salmonella In Silico* Typing Resource (SISTR): An Open Web-Accessible Tool for Rapidly Typing and Subtyping Draft *Salmonella* Genome Assemblies

**DOI:** 10.1371/journal.pone.0147101

**Published:** 2016-01-22

**Authors:** Catherine E. Yoshida, Peter Kruczkiewicz, Chad R. Laing, Erika J. Lingohr, Victor P. J. Gannon, John H. E. Nash, Eduardo N. Taboada

**Affiliations:** 1 National Microbiology Laboratory at Guelph, Public Health Agency of Canada, Guelph, Ontario, Canada; 2 National Microbiology Laboratory at Lethbridge, Public Health Agency of Canada, Lethbridge, Alberta, Canada.; University of Osnabrueck, GERMANY

## Abstract

For nearly 100 years serotyping has been the gold standard for the identification of *Salmonella* serovars. Despite the increasing adoption of DNA-based subtyping approaches, serotype information remains a cornerstone in food safety and public health activities aimed at reducing the burden of salmonellosis. At the same time, recent advances in whole-genome sequencing (WGS) promise to revolutionize our ability to perform advanced pathogen characterization in support of improved source attribution and outbreak analysis. We present the *Salmonella In Silico* Typing Resource (SISTR), a bioinformatics platform for rapidly performing simultaneous *in silico* analyses for several leading subtyping methods on draft *Salmonella* genome assemblies. In addition to performing serovar prediction by genoserotyping, this resource integrates sequence-based typing analyses for: Multi-Locus Sequence Typing (MLST), ribosomal MLST (rMLST), and core genome MLST (cgMLST). We show how phylogenetic context from cgMLST analysis can supplement the genoserotyping analysis and increase the accuracy of *in silico* serovar prediction to over 94.6% on a dataset comprised of 4,188 finished genomes and WGS draft assemblies. In addition to allowing analysis of user-uploaded whole-genome assemblies, the SISTR platform incorporates a database comprising over 4,000 publicly available genomes, allowing users to place their isolates in a broader phylogenetic and epidemiological context. The resource incorporates several metadata driven visualizations to examine the phylogenetic, geospatial and temporal distribution of genome-sequenced isolates. As sequencing of *Salmonella* isolates at public health laboratories around the world becomes increasingly common, rapid *in silico* analysis of minimally processed draft genome assemblies provides a powerful approach for molecular epidemiology in support of public health investigations. Moreover, this type of integrated analysis using multiple sequence-based methods of sub-typing allows for continuity with historical serotyping data as we transition towards the increasing adoption of genomic analyses in epidemiology. The SISTR platform is freely available on the web at https://lfz.corefacility.ca/sistr-app/.

## Introduction

Food-borne salmonellosis is an important public health concern worldwide. For nearly 100 years serotyping has been the gold standard for *Salmonella* classification. As the virulence, host range, and epidemiology of *Salmonella* isolates can be serotype-specific, this classification scheme has been essential for use in human disease surveillance activities and outbreak investigations [1,2]. Currently, *Salmonella* isolates are classified using the White-Kauffmann-Le Minor (WKL) scheme based on serological detection of expressed O (somatic) and H (flagellar) antigens [3]. This scheme is utilized by public health organizations worldwide. Despite its usefulness, serology-based serotyping is labour-intensive, expensive and can take several days to complete.

In recent years, several molecular methods for *Salmonella* typing have emerged in an effort to increase throughput and discriminatory power while reducing costs associated with serotyping [4–12]. With these goals in mind, and in an effort to maintain the identification of a serovar and antigenic formula consistent with WKL, we developed and validated the *Salmonella* Genoserotyping Array (SGSA) [13,14]. Genoserotyping, or DNA-based serotyping, relies on the detection of genetic differences in somatic and flagellar determinants for antigen and serovar prediction. Differences between serogroups are due to variations in the *rfb* region, primarily in the O-antigen flippase (*wzx*) and polymerase (*wzy*) genes, which are considered serogroup-specific [15]. H antigen differences are related to variation in the genes encoding the flagellin structure, *fli*C and *flj*B, which have highly conserved 5’ and 3’ ends and highly variable centre regions [16,17]. The SGSA was developed and validated as a rapid molecular method for non-serological antigen-based serotyping of the most commonly reported *Salmonella* serovars, offering preserved continuity with historical surveillance data.

In addition to genoserotyping approaches, a large number of molecular sub-typing methods including pulsed field gel electrophoresis (PFGE) and Multi Locus Sequence Typing (MLST) have been developed to provide additional discrimination in the analysis of several prevalent *Salmonella* serovars. The use of multiple diagnostic techniques to characterize an isolate causes delays in identification, ultimately impeding progress during outbreak investigations and other public health interventions. Despite the use of molecular sub-typing approaches, many clinically relevant *Salmonella* serovars including Enteritidis have limited genetic diversity and therefore require even greater discrimination.

The deployment of whole genome sequencing (WGS) in the context of public health investigations is becoming increasingly feasible, with the cost and time required for WGS of a bacterial pathogen soon becoming practical in public health laboratories [18–21]. In contrast to the current gold-standard techniques for characterizing bacterial pathogens, which assess only a small proportion of genetic information, sequence data from the entire bacterial genome enables the assessment of many biological attributes of an isolate simultaneously [22]. Moreover, the increasing availability of analytical approaches for whole genome-based sub-typing will continue to fuel the adoption of genomics in the context of epidemiological investigations [23–26]. A current challenge facing the global public health community is balancing the adoption of WGS-based approaches while still relying on the well-established methods that are the cornerstone of molecular surveillance programs. The transition towards an era of ‘genomic epidemiology’ will require linking of newly sequenced genomes with the isolates and traditional sub-typing data in databases such as PulseNet [27] and MLSTdb [28] in order to preserve relevant epidemiological information for public health investigations.

In this study, we present the *Salmonella In Silico* Typing Resource (SISTR), an open web-accessible tool that allows users to upload minimally processed *Salmonella* draft genome assemblies and to perform rapid *in silico* molecular typing. In addition to providing serovar prediction using a genoserotyping approach that has been previously developed and validated by our group [13,14], this resource integrates several additional sequence-based typing analyses that include MLST [29], ribosomal MLST (rMLST) [30], and core genome MLST (cgMLST) [23,24] into a single web-based tool that incorporates a database comprised of more than 4,000 genome sequences, representing 246 *Salmonella* serovars. We present here how information derived from cgMLST to supplement genoserotyping analysis can be used to increase the accuracy of *in silico* serovar prediction. Genoserotyping and advanced molecular typing based on WGS analysis provides the advantage of generating legacy data while also paving the way towards more advanced forms of *Salmonella* isolate characterization.

## Methods

### SISTR server implementation

The SISTR platform consists of a Python (v2.7.9) server application (www.python.org), communicating with a PostgreSQL (v9.4.1) database (www.postgresql.org) and a Reagent (v0.5.0) ClojureScript (v0.0–3269) web application (http://holmsand.github.io/reagent; https://github.com/clojure/clojurescript). The server app is implemented in Python using the Flask web micro web framework (v0.10.1; http://flask.pocoo.org) and communicates with the PostgreSQL database using SQLAlchemy (v1.0.2; http://www.sqlalchemy.org). The server app exposes a REST API through which the user-facing SISTR web application sends and receives data [31]. A Redis key-value datastore (v3.0.1) is used for caching data requested from the server app (http://redis.io). A Celery-distributed task queue (v3.1.18) is used for asynchronously running tasks such as *in silico* analyses on user uploaded genomes (http://www.celeryproject.org).

### *In silico* subtyping analyses

*In silico* derivation of molecular subtyping data in the SISTR platform is performed using the “Microbial *In silico* Typing” (MIST) engine. An analytical platform developed by our group, MIST allows users to simulate a range of molecular assays on draft genome sequence data [32].

#### Analysis using pre-established sequence typing schemes

Existing sequence typing schemes were obtained from published literature. These included the Multi-Locus Sequence Typing (MLST) scheme for *Salmonella* previously described by Achtman et al. [33], and the ribosomal MLST (rMLST) scheme described by Jolley et al. [30]. Both assays are incorporated into the SISTR server as MIST-based *in silico* sequence typing assays.

#### *In silico* serovar prediction

The serovar prediction module in the SISTR server utilizes O (somatic) and H (flagellar) antigen and/or serogroup-specific probes previously designed for our *Salmonella* Genoserotyping Array (SGSA), which provides serovar identification for 90% (n = 2,190) of serovars [13,14]. **Antigen identification:** The SGSA probes, which were previously described and validated experimentally [11,13], were incorporated into a MIST-based *in silico* hybridization assay. Our serovar prediction algorithm uses the *in silico* results to create a query based on O serogroup, H1, and H2 antigen gene sequences that is used to identify the serovar based on the antigenic formula [3]. **Serovar identification:** Because draft genome assemblies may generate incomplete data for the antigenic query, the algorithm incorporates logic that allows for partial matching of the antigenic formula. Results with multiple possible serovars use the “phylogenetic context”, whereby the predominant serovar of genomes within the same cgMLST cluster (defined at an 85% profile similarity) is used to identify the most likely serovar. This profile similarity threshold was chosen so as to maximize the proportion of genomes in multi-isolate clusters without adversely affecting the specificity of the correlation between cgMLST cluster and the reported serovar (S1 Fig). In order for evidence from cgMLST to be used for refining a genoserotyping prediction, we used a minimum cgMLST cluster size (n = 4) and a minimum consensus support of 75%. The minimum cluster size was chosen so that a meaningful consensus support (i.e. three out of four members of the cluster) could be derived, given evidence for genomes with incorrect reported serovar in the dataset.

When a unique serovar is identified based on antigen identification, the SISTR serovar prediction pipeline is complete. The phylogenetic context from cgMLST is used for serovar prediction only when it is not possible or incomplete by genoserotyping.

#### Core genome MLST (cgMLST) analysis

For efficient computation of phylogenies we use a method derived from the approach for whole genome MLST (wgMLST) previously described by Sheppard et al. [23] but focusing on core genes, which has been termed ‘core genome MLST’ (cgMLST). ***Salmonella* core genome identification:** To identify core genes in *Salmonella* we sampled a wide cross-section of publicly available genomes from different serovars and subspecies (n = 361), focusing on completed genomes and genome assemblies of highest quality (i.e. N50 > 300,000 bp, fewer than 100 contigs > 500 bp) in a dataset comprised of 584 genomes originally used to test the SISTR *in silico* O and H antigen predictions and the overall serovar prediction logic. For information on the strains used for core genome definition and cgMLST scheme creation please see S1 File. After performing gene prediction on all genomes using Prodigal (v2.60) [34], which yielded a total of 1,619,015 genes, we used CD-HIT (v4.6.1) [35,36] to identify a non-redundant set of gene clusters representing likely orthologous genes (n = 17,867). A representative nucleotide sequence from each unique orthologous gene was then homology searched against the 361 genomes assemblies using BLASTN (v2.2.28) [37]. A set of 3,496 core genes was found in all genomes. **cgMLST assay design:** To avoid potential ambiguities in cgMLST allele assignment we constructed a multiple sequence alignment for each core gene using MAFFT (v7.147) [38] in order to identify those genes that contained full-length coverage and 0 indels across the entire length of the alignment among the 361 genomes analyzed. Three hundred and thirty core genes passed these strict selection criteria and were used in the design of the cgMLST assay currently implemented in the SISTR server as a MIST-based *in silico* sequence typing assay. This cgMLST scheme (cgMLST330) is a prototype used to test a range of cgMLST applications in the SISTR platform; allelic data for the 330 loci included in the scheme are available for download (http://lfz.corefacility.ca/sistr-mist-assays/). **cgMLST-based phylogenetic reconstruction:**

The pairwise cgMLST similarity between any two genomes is computed as the proportion of variant alleles between the two genomes divided by the total number of loci, corrected for missing loci in either genome. For hierarchical clustering analysis, the complete matrix of pairwise distances is clustered using the *fastcluster* Python module (http://www.jstatsoft.org/v53/i09/) [39]. For minimum spanning tree analysis distance matrices are clustered using Kruskal’s algorithm using the *networkx* Python module (https://networkx.github.io/). Computations occur in real-time and on-demand.

#### Data visualizations

The basic interface for displaying predicted *in silico* typing data is in the form of a user-customizable interactive table rendered using SlickGrid (https://github.com/mleibman/SlickGrid/wiki) with user-defined fields and custom sorting. Table filtering is performed using Selectize.js (http://brianreavis.github.io/selectize.js/). The predicted serovar and antigenic formula are displayed in the WKL format. The SISTR platform uses interactive visualizations for minimum-spanning trees, phylogenetic trees, bar charts and pie charts, implemented using D3.js (v3.5.5; http://d3js.org) to facilitate the examination of trends in the phylogenetic, geospatial, and temporal distribution of genomes. Geographical visualizations are implemented using Leaflet.js (v0.7.3; http://leafletjs.com), and D3.js for metadata pie charts. Existing epidemiological metadata and derived *in silico* typing metadata can be projected onto all visualizations.

### Validation of SISTR analyses

#### *Salmonella* sequences

*Salmonella* whole genome sequences used in this study (n = 4,291) were obtained from the public repositories of WGS data at the National Center for Biotechnology Information (NCBI). These included 578 fully assembled genomes from NCBI Assembly (http://www.ncbi.nlm.nih.gov/assembly/), and unassembled genomes (n = 3,713) downloaded from the NCBI SRA repository (http://www.ncbi.nlm.nih.gov/sra). Unassembled genomes were assembled using SPAdes (v3.1.1) [40]. Assembly metrics (e.g. N50, largest contig, number of contigs > 1000 bp, etc) were computed using QUAST (v2.3) [41] and visualized using Qviz (https://lfz.corefacility.ca/shiny/qviz/), an interactive web-based tool developed in our group for visualizing assembly metrics for large numbers of genomes. Of the genome sequences analyzed, some had to be removed from the final analysis due to missing or incomplete serovar information in the supplied metadata (n = 79). Other genomes were removed due to poor assembly metrics (n = 8). A small number of genomes appeared to be non-*Salmonella* (n = 12) and were also removed from the analysis.

#### Assessment of antigen and serovar predictions and comparison with reported serovar results

For assessment and validation of the full SISTR prediction pipeline, the complete set of 4,291 genome sequences was analyzed. The accuracy of predictions were computed based on the proportion of concordant calls between “reported” serovar, which was based on the metadata supplied with the genome sequence data, and the “predicted” serovar based on our *in silico* prediction methodology. To further test the specificity of various *in silico* analyses, the prediction pipeline was also challenged with non-*Salmonella* (i.e. *Escherichia coli*) genomes.

## Results

### Analysis of predicted serovar calls

**Fig 1:**
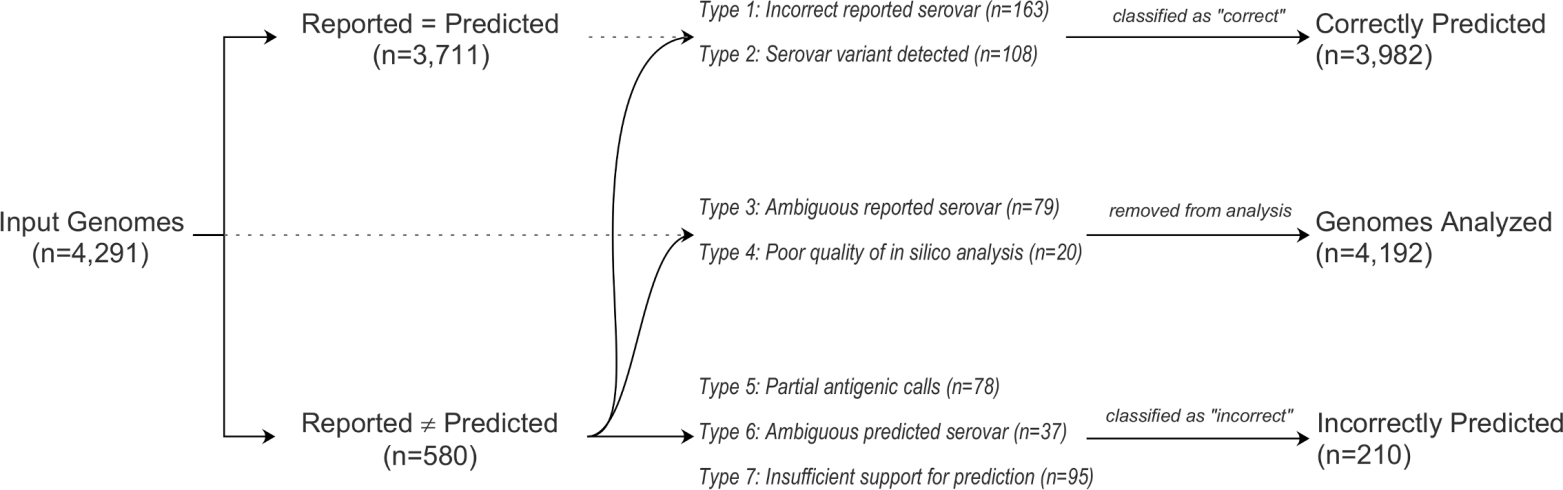
Analysis of sources of error in serovar predictions on a set of 4,291 *Salmonella enterica* draft genome sequences analyzed using the SISTR platform. The contribution of various types of errors contributing to observed differences between “reported” and “predicted” serovars was tabulated. From a total of 4,192 genomes retained for the analysis, 3,982 genomes had correct serovar predictions (94.99%).

A preliminary examination of *in silico* serovar prediction results revealed that 3,707 of 4,291 genomes analyzed (i.e. 86.4%) had a prediction that matched the reported serovar based on the information extracted from the metadata supplied for that genome. The remaining 584 cases showed discrepancies between the reported and predicted serovar and were further examined to assess factors contributing to mismatches (Fig 1 and S1 File).

**Fig 2:**
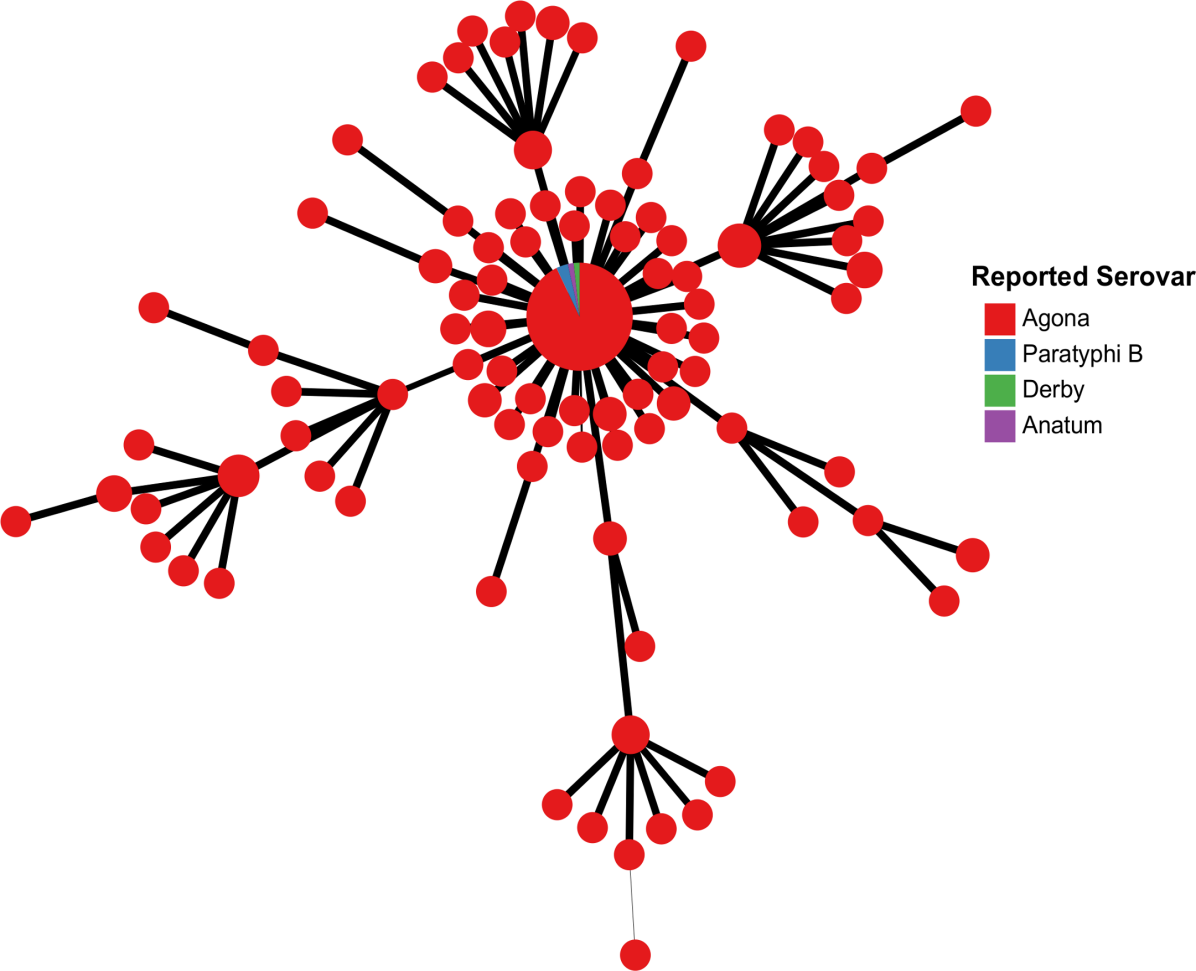
*In silico* serovar prediction identifies instances of *Salmonella* genomes with incorrect reported serovar information. Among a large cgMLST330 cluster of genomes of reported serovar Agona were found four genomes of different reported serovar (Paratyphi B, n = 2; Anatum, n = 1; Derby, n = 1). Upon closer inspection, the serovar prediction for these genomes was found to be Agona, consistent with the underlying cgMLST330 data.

Seven types of errors were observed: **Type 1: An incorrect reported serovar.** A very high concordance was observed between cgMLST cluster membership and serovar. In a large number of cases (n = 152), evidence from cgMLST strongly suggested that the serovar supplied in the metadata for the genome in question was incorrect. In these cases, the genome was part of a cgMLST cluster in which most or all of the other members had a reported serovar that matched the predicted serovar (Fig 2). For the purposes of assessing the accuracy of prediction, these cases were reclassified as serovar matches. **Type 2: A serovar variant detected.** In a significant proportion of cases (n = 108), the reported serovar was a known variant of the predicted serovar (e.g. Cholerasuis var. Kunzendorf [42]). These serovar variants are generally defined by the expression of variable individual O antigenic factors, and additional phenotypic traits. The genoserotyping is currently limited to the detection of somatic serogroups, which are a defined group of individual O antigenic factors as detailed by WKL. The presence and expression of additional individual O antigens is occasionally required to differentiate serotypes or variants. SISTR does not identify or report serovar variants requiring biochemical or sub-speciation tests for full characterization. SISTR infers a serovar which may require additional phenotypic information for final characterization in the following circumstances: 1) When multiple serovars have the same antigenic formula in the White-Kauffmann-Le Minor scheme; 2) When a partial antigenic formula is identified; and 3) For exceptions (e.g. Choleraesuis/Paratyphi C/Typhisuis/Chiredzi, Sendai/Miami, Paratyphi B/Paratyphi B var. Java). It may be possible, however, for a future implementation of the SISTR analysis pipeline to incorporate predictions for any defining phenotypic traits for which genetic determinants are known or are identified in the future. In addition, the high level of discrimination provided by cgMLST may be used in a future implementation of SISTR to identify serovar variants. For the purposes of assessing the accuracy of prediction, these cases were reclassified as serovar matches. **Type 3: Ambiguous reported serovar.** This category represented cases (n = 79) in which missing or ambiguous metadata made it impossible to ascertain the reported serovar. For the purposes of assessing serovar prediction accuracy these cases were removed from the dataset. **Type 4: Poor quality of cgMLST data.** In 20 cases, cgMLST data was of poor quality, with 30 or more missing or incomplete loci. A further examination of these cases revealed that in 8 instances the genomes had assembly metrics typical of poor quality assemblies. In the remaining 12 cases the genome assemblies were of sufficient quality for successful *in silico* analysis but evidence from sequence homology suggested that these were not of *Salmonella* origin but genomes from other species including *Escherichia coli* and *Citrobacter freundii*. For the purposes of assessing serovar prediction accuracy, all genomes with errors of Type 4 were removed from the dataset. **Type 5: Partial antigenic calls.** In a number of cases (n = 93) a serovar prediction could not be made due to missing data for one or more antigens. Although in many cases one or two antigens were predicted and matched those of the reported serovar, determination of the serovar was not possible. As with Type 2 errors, the identification of individual O antigen factors may be required for serovar identification. **Type 6: Ambiguous predicted serovar.** In a small number of cases (n = 37), the prediction could not be narrowed down to a single serovar, typically for serovars from different subspecies that share the same antigenic formula. These cases often represented genomes from lineages with insufficient examples in the dataset, which precluded the use of contextual phylogenetic information from cgMLST to narrow down the likely serovar. **Type 7: Insufficient support for predicted serovar.** Although cases in which genomes appeared to have an incorrect reported serovar (i.e. errors of Type 1) are prominent in the dataset, in some cases (n = 94) there was insufficient evidence from cgMLST in support for the predicted serovar to supersede the reported serovar.

### Effect of genome assembly quality on serovar prediction

**Fig 3:**
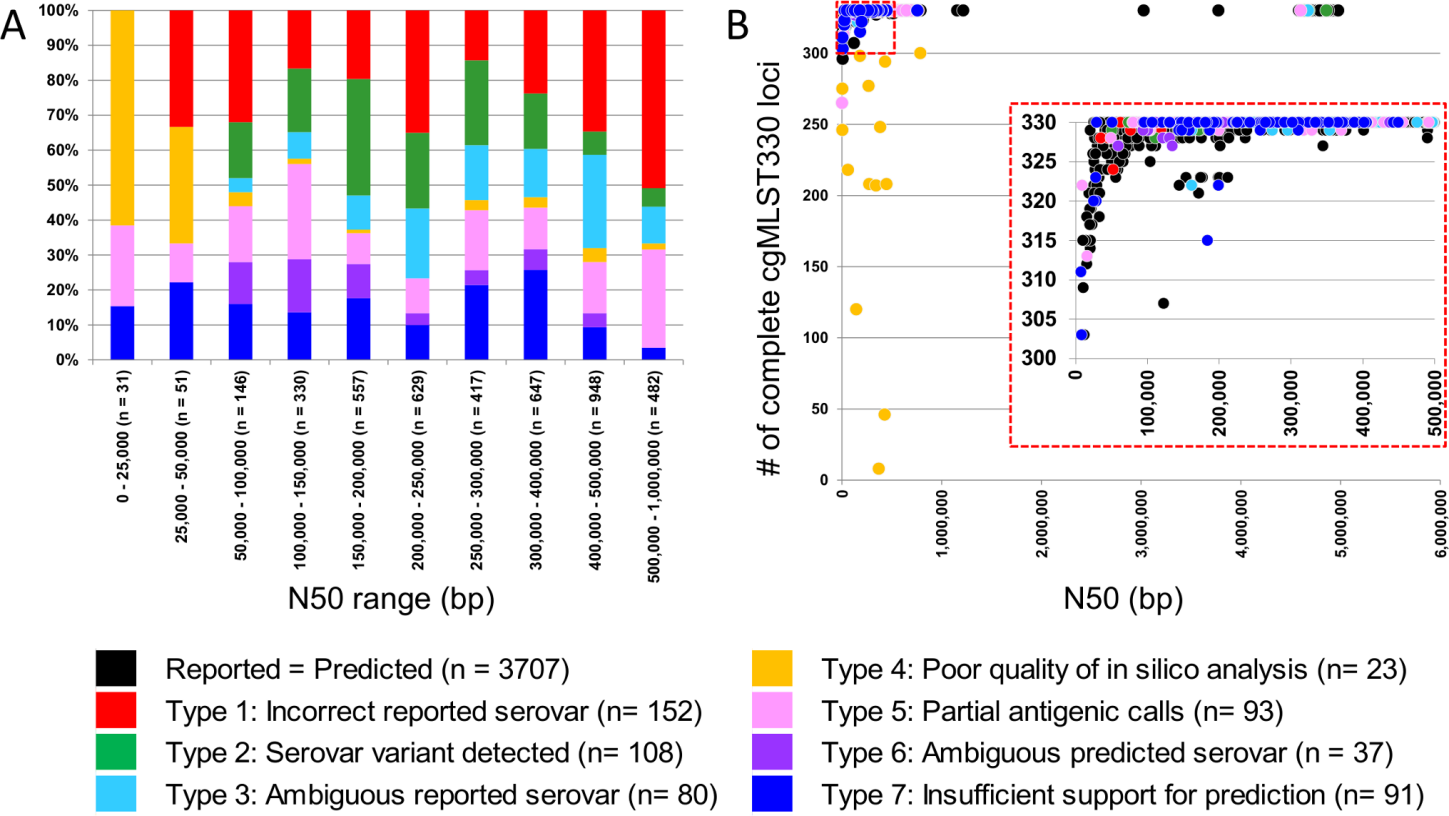
The SISTR serovar prediction logic can robustly yield accurate predictions for a range of genome qualities. (A) The relative proportion of various error types does not change appreciably as a function of the N50 assembly quality parameter. Type 4 errors, which are related to poor cgMLST330 metrics, are only observed among genomes with lowest N50 values. (B) Although large numbers of missing cgMLST330 loci affect serovar prediction, as observed with errors of Type 4, accurate predictions were also made for genomes with as few as 296 complete cgMLST330 loci.

Because of the potential for the quality of WGS data to affect *in silico* calls, we examined the effect of genome sequence quality upon the accuracy of serotype prediction. To this end, we examined the rate for the various errors types described above as a function of the N50 parameter (Fig 3A). In general, we observed no significant differences in error type distribution across the range of N50 values. One notable exception was a much larger contribution of errors of Type 4, which are defined by poor cgMLST data, among genomes with the worst N50 values. The completeness of cgMLST330 data is used in SISTR for assessing genome assembly quality; we thus also examined the various error types as a function of complete cgMLST330 loci (Fig 3B). The genomes with fewest cgMLST loci did not yield successful predictions although, as previously mentioned, this also included 12 genomes that do not appear to be of *Salmonella* origin. Among the 3,967 genome assemblies with correct predictions, which included both errors of Type 1 (incorrect reported serovar) and Type 2 (serovar variant detected), the median N50 was 303,132 bp, however, 71 assemblies had N50 values of less than 50,000 bp. Similarly, although most of the genomes with correct predictions had a complete set of cgMLST330 loci, we observed accurate predictions for genomes with as few as 296 complete loci. Thus, although high quality assembly metrics, either in the form of a high N50 or complete cgMLST330 loci, yielded a high accuracy of prediction, the SISTR prediction pipeline is robust enough to yield successful predictions for assemblies of lesser quality. Of note, among assemblies with N50 values of less than 50,000 bp, 86.6% (71/82) were accurately predicted.

### Analysis of serovar prediction accuracy

We analyzed the concordance between the set of genomes from a given reported serovar and the dominant predicted serovar as a proxy for “serovar prediction accuracy”. The prediction accuracy was calculated by reclassifying 260 genomes as correct predictions, including those with errors of Type 1 (incorrect reported serovar; n = 152) and Type 2 (serovar variant detected; n = 108), and by removing 99 genomes from the analysis, including those with errors of Type 3 (ambiguous reported serovar; n = 79) and Type 4 (poor quality of cgMLST data; n = 20), since their accuracy of prediction could not be assessed. A total of 3,967 genomes were accurately predicted out of a total of 4,191 genomes included in this analysis, for a global prediction accuracy of 94.9%.

**Fig 4:**
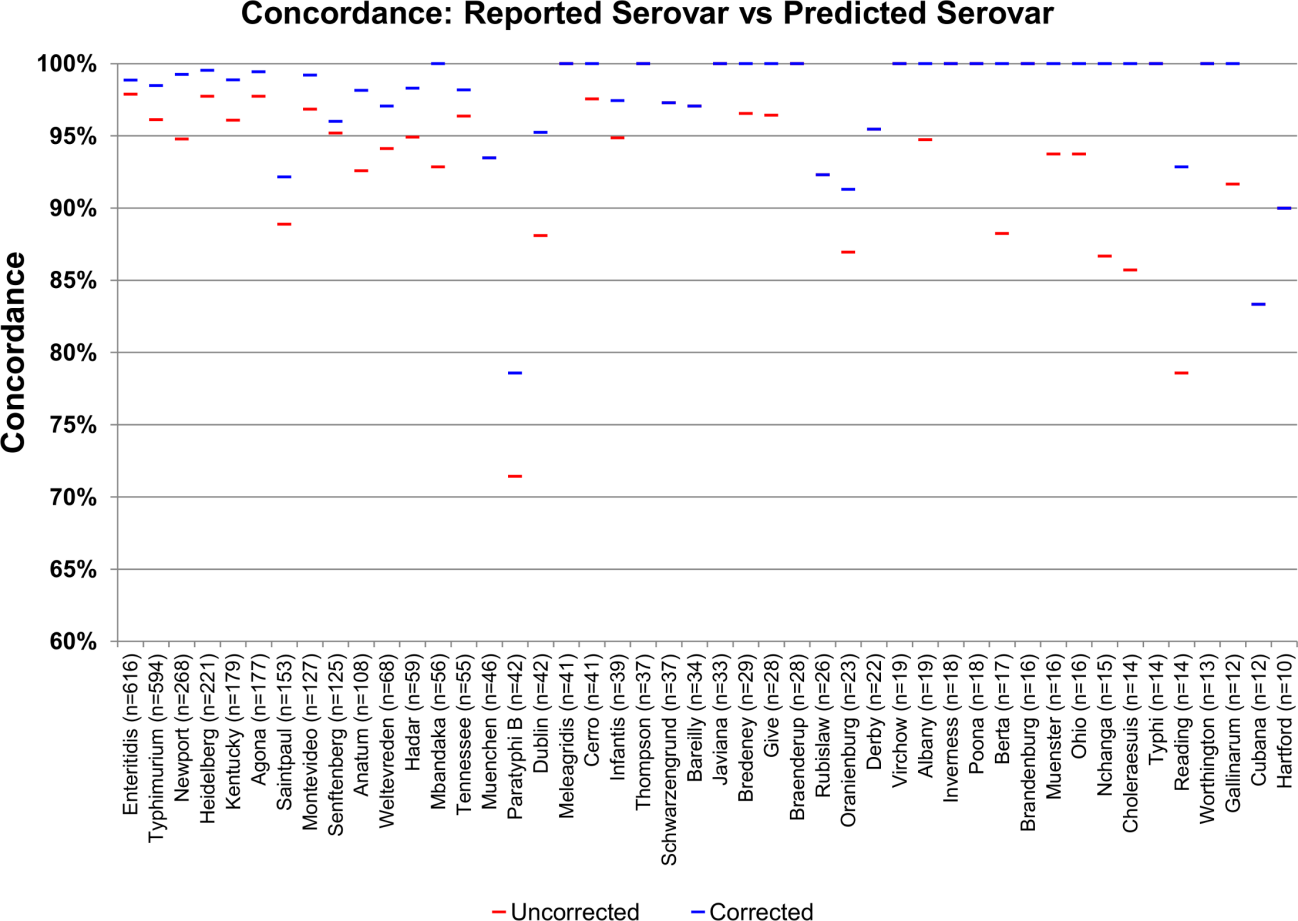
A high accuracy was observed for the SISTR serovar prediction pipeline. The prediction accuracy was assessed for serovars with 10 or more genome representatives and based only on genomes with metadata of sufficient quality to enable extraction of serovar information and those with high cgMLST data quality (n = 4,188). Accuracy was computed based on concordance between reported and predicted serovars. The “uncorrected” prediction accuracy, which is based on the original set of input genomes (n = 4,291) is shown in red. A “corrected” prediction accuracy, which is based on reclassification of genomes with Type 1 and 2 errors, and removal of genomes with Type 3 and 4 errors, is shown in blue. (note: where distinct corrected and uncorrected concordance values are not observable, both values are identical).

The accuracy of prediction was also assessed on a per serovar basis. For serovars with a minimum of four genomes in the dataset, 79 of 84 had at least a 75% concordance between reported and predicted serovar. Among serovars with ten or more genomes, 35 of 46 had a concordance of 97% or higher (Fig 4) and only two serovars, Paratyphi B and Cubana (n = 42 and n = 12, respectively), had a concordance below 90%.

**Fig 5:**
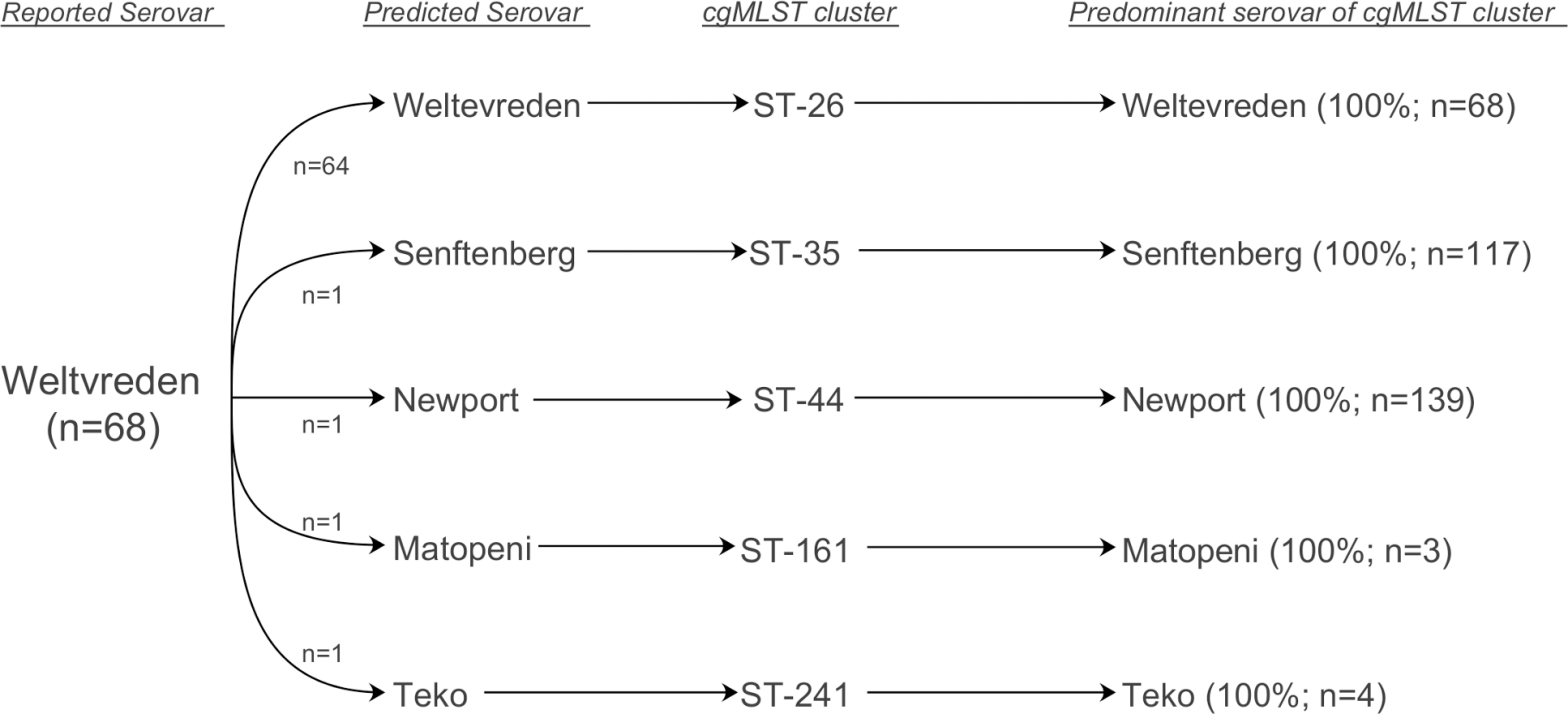
Differences between reported and predicted serovars for Weltevreden genomes are due to an incorrect reported serovar. While a large majority of genomes analyzed in the SISTR server were predicted as Weltevreden (n = 64), the remaining four genomes were predicted to have different serovars; these predictions matched the predominant serovar of their corresponding cgMLST cluster. The percent concordance between cgMLST and serovar and cgMLST cluster size are shown in parentheses.

In general, the trend observed was that for a set of genomes from a given reported serovar a dominant predicted serovar was observed, along with a smaller group of genomes with predicted serovars differing from the reported serovar. In most cases, these genomes belonged to cgMLST clusters strongly associated with the predicted serovar, suggesting that the reported serovar was incorrect. For example, while 64 of 68 reported Weltevreden genomes had a matching prediction, the remaining 4 genomes had predictions that differed from the reported serovar but that matched the predominant serovar corresponding to their respective cgMLST cluster (Fig 5).

### Analysis of cgMLST cluster and serovar concordance

**Fig 6:**
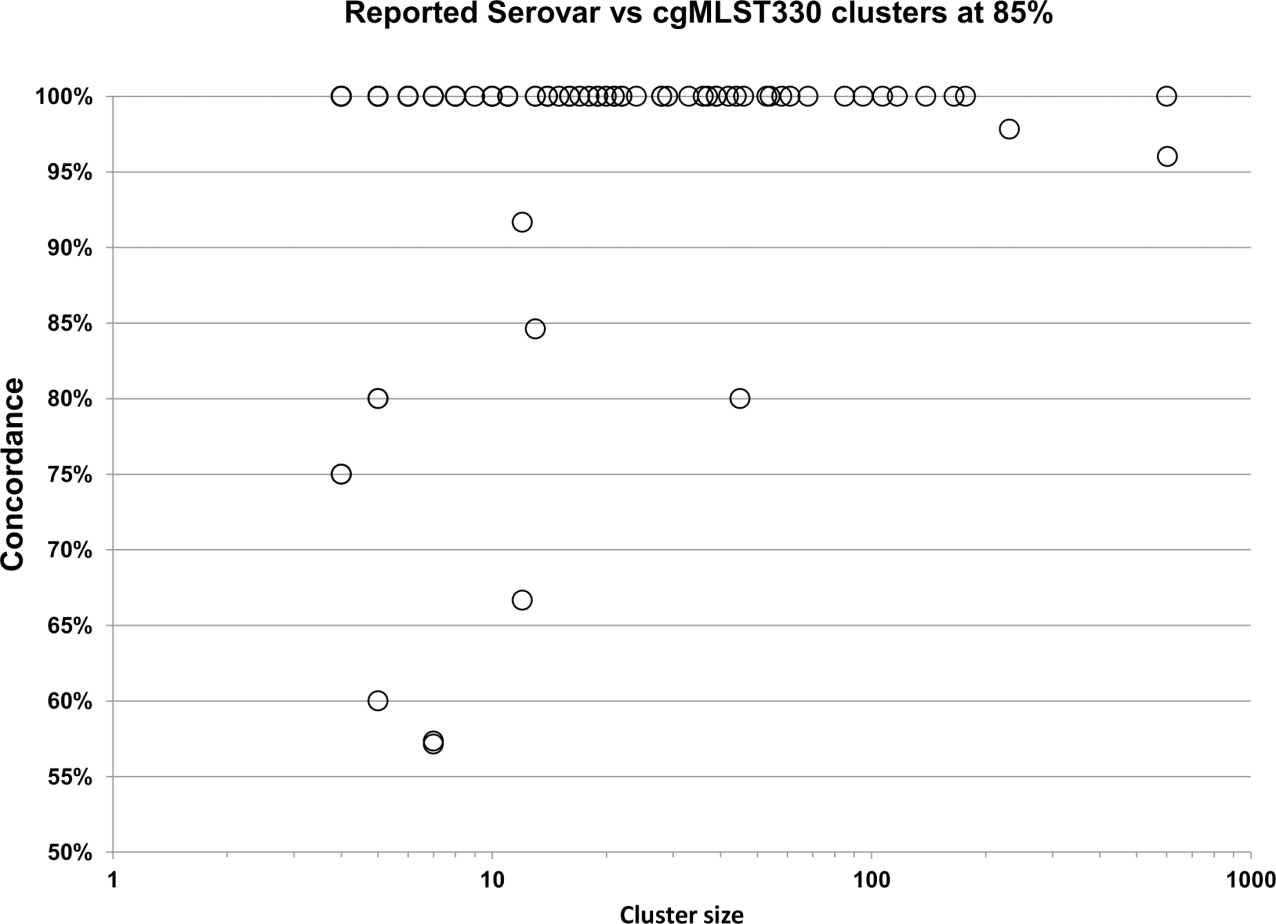
A high level of concordance observed between cgMLST cluster and serovar. The concordance was based on the proportion of genomes in a cgMLST cluster that belonged to the predominant predicted serovar in the group. The cgMLST clusters were defined at a similarity threshold of 85%; only clusters with four or more members are shown.

A close relationship was observed between a genome’s serovar and its cgMLST cluster. In order to systematically examine this relationship, the level of concordance between each cgMLST cluster and the dominant predicted serovar in that cluster was analyzed. Of cgMLST clusters with a minimum of four genomes in the dataset, 111 of 116 had at least 75% concordance between cgMLST cluster and dominant serovar, with a global concordance of 96.4%. Moreover, among cgMLST clusters with ten or more genomes, 52 of 58 had a concordance of 100% (Fig 6) and only three had a concordance below 90%.

**Fig 7:**
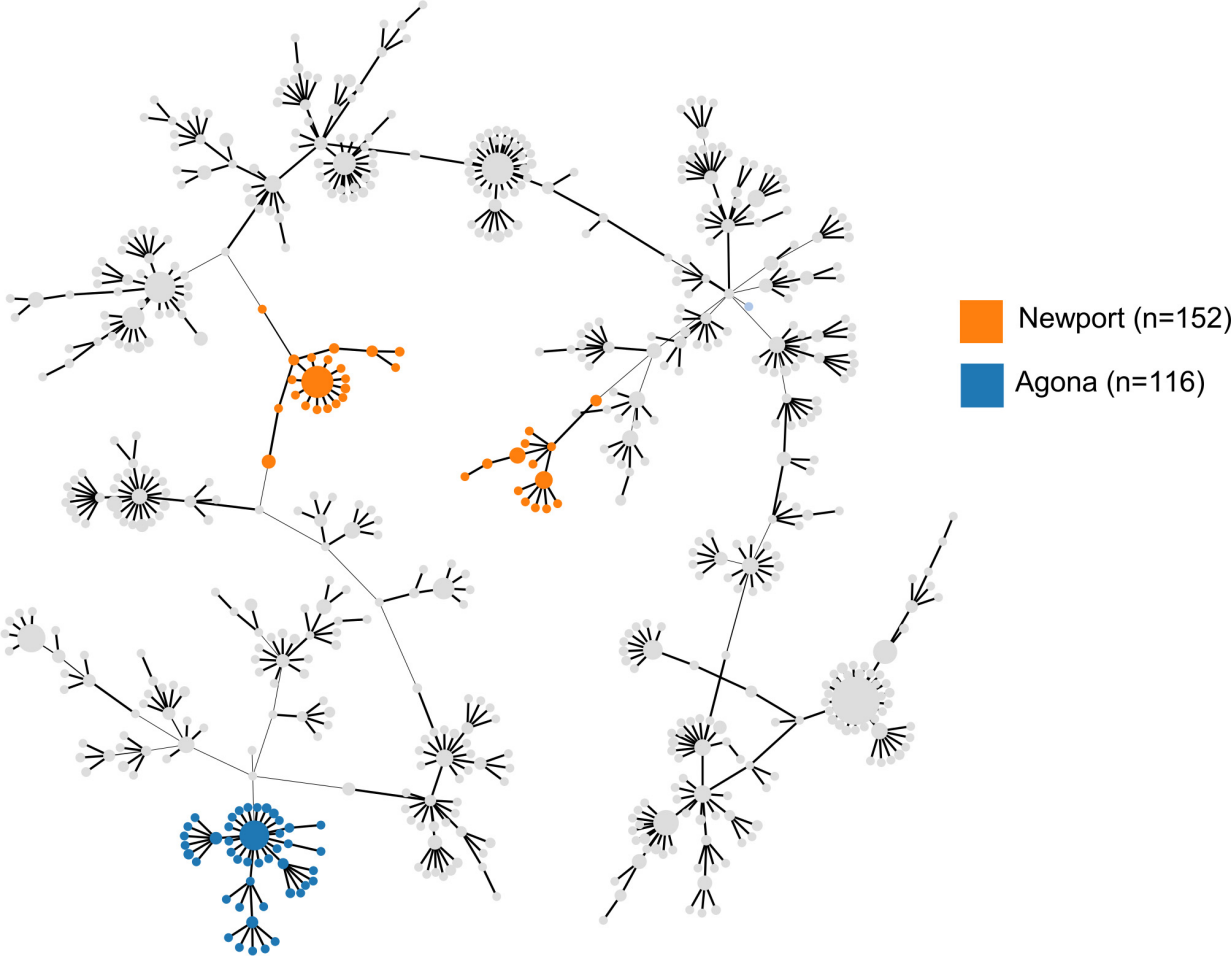
Evidence from cgMLST supports the polyphyletic origin of *Salmonella* Newport. Minimum Spanning Tree visualization of cgMLST phylogeny for a set of *Salmonella enterica* genomes (n = 2,002) created in the SISTR server. The predicted serovar for Newport and Agona genomes has been projected onto the tree to highlight the contrast between a polyphyletic serovar (Newport) and a monophyletic serovar (Agona).

The concordance levels observed would suggest that in most cases there is a one to one correlation between a given cgMLST cluster and a particular serovar. However, in a number of cases, one serovar could be associated with a small number of cgMLST clusters, with most of the genomes belonging to a single dominant cgMLST cluster. In most cases, this was owing to the much higher discriminatory power of cgMLST; 422 clusters at 85% profile similarity and 2,405 distinct cgMLST profiles were observed among the 4,191 genomes in the dataset. However, for genomes from certain serovars (e.g. Newport), there was no single dominant cgMLST cluster association, with genomes evenly dispersed among several clusters. Moreover, associated cgMLST clusters could be quite genetically distinct, suggesting a polyphyletic origin for certain serovars through the horizontal transfer of serovar determinants among unrelated lineages in the *Salmonella enterica* population (Fig 7). These data provide strong support for previous observations that have been made using MLST [33].

## Discussion

In this study, we present the *Salmonella In Silico* Typing Resource (SISTR), an open web-accessible analytical platform that allows users to upload minimally processed *Salmonella* draft genome assemblies and to perform rapid and simultaneous *in silico* molecular typing using a number of complementary approaches.

In addition to providing genoserotyping-based serovar prediction [13,14], this resource integrates several additional sequence-based typing analyses that include MLST [29], rMLST [30], and cgMLST [23,24] into a single web-based tool. The platform currently incorporates a database comprised of more than 4,000 genome sequences, capitalising on the growing body of *Salmonella* WGS data that is becoming publicly available and allowing users to place their genome-sequenced isolates in a greater epidemiological and phylogenetic context. The SISTR platform also includes an expanding set of metadata-driven visualizations that allow users to examine the phylogenetic, geospatial, and temporal relationships between genome-sequenced isolates based on any number of biological and epidemiological attributes.

The SISTR platform provides serovar prediction using a genoserotyping approach that has been previously developed and extensively validated by our group [13,14], which the serovar prediction pipeline complements with supporting evidence to make informed predictions when genoserotyping cannot narrow down the prediction to a single possible serovar or when antigenic prediction results are of poor quality. Of the *Salmonella* genomes analysed as part of our validation efforts (n = 4,291), there was a 94.6% overall serovar prediction accuracy. It is worth noting that this value is a conservative estimate, as cgMLST provided strong evidence for serovar predictions for a further 103 genomes, 60 of 80 genomes with errors of Type 3 (ambiguous reported serovar) and 43 of 94 genomes with errors of Type 5 (partial antigenic call). Moreover, errors of Type 7 (insufficient support for predicted serovar) are likely to be overestimated since one of the criteria used for identifying probable cases with incorrect metadata (i.e. errors of Type 1) was a minimum cgMLST cluster size of four; any genomes in smaller clusters, 51 of 96 genomes with errors of Type 7, were not considered for cgMLST correction.

A key analysis performed in the SISTR is cgMLST, a gene-by-gene approach to genome-based phylogenetic analysis derived from the approach for whole genome MLST (wgMLST) previously described by Sheppard et al. [23] but focusing on core genes. In the context of serovar prediction, although genoserotyping results take precedence in the analysis, cgMLST results are used to confirm genoserotyping predictions and to provide assistance in cases when antigenic data are incomplete, for example due to incomplete WGS data or due to the lack of suitable probes for certain antigens. It is important to note that an approach that uses both lines of evidence is superior to either method alone, as there is not a full correlation between serovar and underlying genetic similarity, which has important implications in terms of surveillance and epidemiological investigations.

In addition to a role in serovar prediction, results from cgMLST are used in the SISTR platform in two other contexts. In the first, the quality of the WGS data is assessed during data upload by the user in the form of complete, partial, and missing cgMLST loci. This metric provides intuitive feedback to the user in cases of aberrant *in silico* typing results, which may be due to low WGS data quality or non-*Salmonella* WGS data. In the second application, cgMLST data is used to generate genetic similarity estimates for phylogenetic analysis, which allows users to examine the phylogenetic distribution of uploaded genomes using the over 4,000 genomes currently used to populate the SISTR database as a frame of reference. The cgMLST scheme currently used in the SISTR platform is based on a robust set of 330 core genes identified through a rigorous comparative genomic analysis based on a set of the best quality genome assemblies in the dataset. This cgMLST scheme (cgMLST330) is a prototype that we have used to test the three basic applications of cgMLST data in the SISTR analytical pipeline: serovar prediction, genome QC, and phylogenetic analysis. There are current efforts by the international community towards the development of a standardized cgMLST scheme for *Salmonella*. A future implementation of the SISTR server will also include this scheme.

An important finding from the validation of our serovar prediction pipeline was how information derived from cgMLST could be used to increase the accuracy of *in silico* serovar prediction and used this approach to identify genome-sequenced isolates in the public domain with what appears to be incorrect reported serovar information. The SISTR platform would not be possible without the *Salmonella* WGS data contributed to the public domain. At the same time, our results highlight the importance that must be placed on the development of stringent metadata standards, which are necessary for these data to be of maximum utility to the global community. Greater effort should be placed towards the development of tools to facilitate the curation of metadata, which should help ease the burden on groups sharing their WGS data in public repositories.

## Conclusions

*Salmonella enterica* remains an important public health concern worldwide, with laboratories relying heavily on the White-Kauffman-LeMinor serotyping scheme as a primary means of *Salmonella* classification. At the same time, it is widely acknowledged that serotyping and other current means of *Salmonella* subtyping often lack the specificity and discriminatory power required in the context of public health surveillance and epidemiologic investigations. With recent advances in Next Generation Sequencing, there is now significant momentum towards the increasing adoption of WGS as a primary means of isolate characterization. This is due to the potential for a single method to replace the multiple approaches currently used, including serotyping and various molecular subtyping methods, with high resolution genomic data generated at a reduced cost and decreased turn-around-time.

Rapid *in silico* analysis of minimally processed *Salmonella* draft genome assemblies in web-based tools such as the SISTR platform provides a powerful approach for facilitating the integration of WGS-based analyses towards epidemiology and public health. This analytical platform capitalizes on previous work by our group on sequence-based serovar identification while also performing advanced sequence typing analyses that include a prototype cgMLST method developed as part of this study. Such genome-based analyses represent the primary motivation for the current move towards WGS and we anticipate that the SISTR platform will be easily amenable to additional analyses, with analytical packages for analysis of virulence gene complement and Antimicrobial Resistance (AMR) profiling to be included in a future implementation of the platform. In addition, the inclusion of additional WGS data currently being generated and being made publicly available by the *Salmonella* research community will increase the accuracy of *in silico* analyses.

As this manuscript was being readied for publication, web resources for serotype prediction from WGS for *Salmonella* and *E*. *coli* were recently described [43,44], which illustrates the continuing relevance of serotyping in the surveillance of these priority pathogens. At the same time, the emergence of SISTR and other similar resources for rapid analysis of WGS data also serves to highlight the need for analytical platforms to facilitate the use of genomics in public health applications. By providing integrated genoserotyping and advanced molecular typing based on WGS-based analyses the SISTR platform provides the advantage of generating legacy data, while also paving the way towards more advanced forms of *Salmonella* isolate characterization as we transition to a ‘genomic epidemiology’ paradigm. Analytical platforms to perform rapid analysis of *Salmonella* genome sequence data using a number of complementary approaches will improve the response capacity of the public health system for the prevention and control of salmonellosis.

## Supporting Information

S1 Fig A cgMLST clustering threshold of 85% similarity balances the proportion of genomes in multi-isolate cgMLST clusters and the serovar specificity of cgMLST clusters. Analyses in this study were performed at a cgMLST clustering threshold of 85% profile similarity. This value maximized the proportion of genomes in clusters with a minimum cluster size of four without adversely affecting the specificity between cgMLST clusters and reported serovar. Cluster size was an important consideration in the analysis since, among cases where reported and predicted serovar did not match, genomes in cgMLST clusters smaller than four members (51 of 96 genomes with errors of Type 7) were not considered for cgMLST correction.
(TIFF)

S1 File Information on genomes (n = 4,291) used to test and validate the SISTR platform. Included are error codes described in Fig 1, along with *in silico* predictions, and genome quality metrics.
(XLSX)
