# Supplementary figures and images for: The Salmonella In Silico Typing Resource (SISTR): An Open Web-Accessible Tool for Rapidly Typing and Subtyping Draft Salmonella Genome Assemblies
Source: PLoS One. 2016 Jan 22;11(1):e0147101. doi: 10.1371/journal.pone.0147101 (PMC4723315; doi:10.1371/journal.pone.0147101)

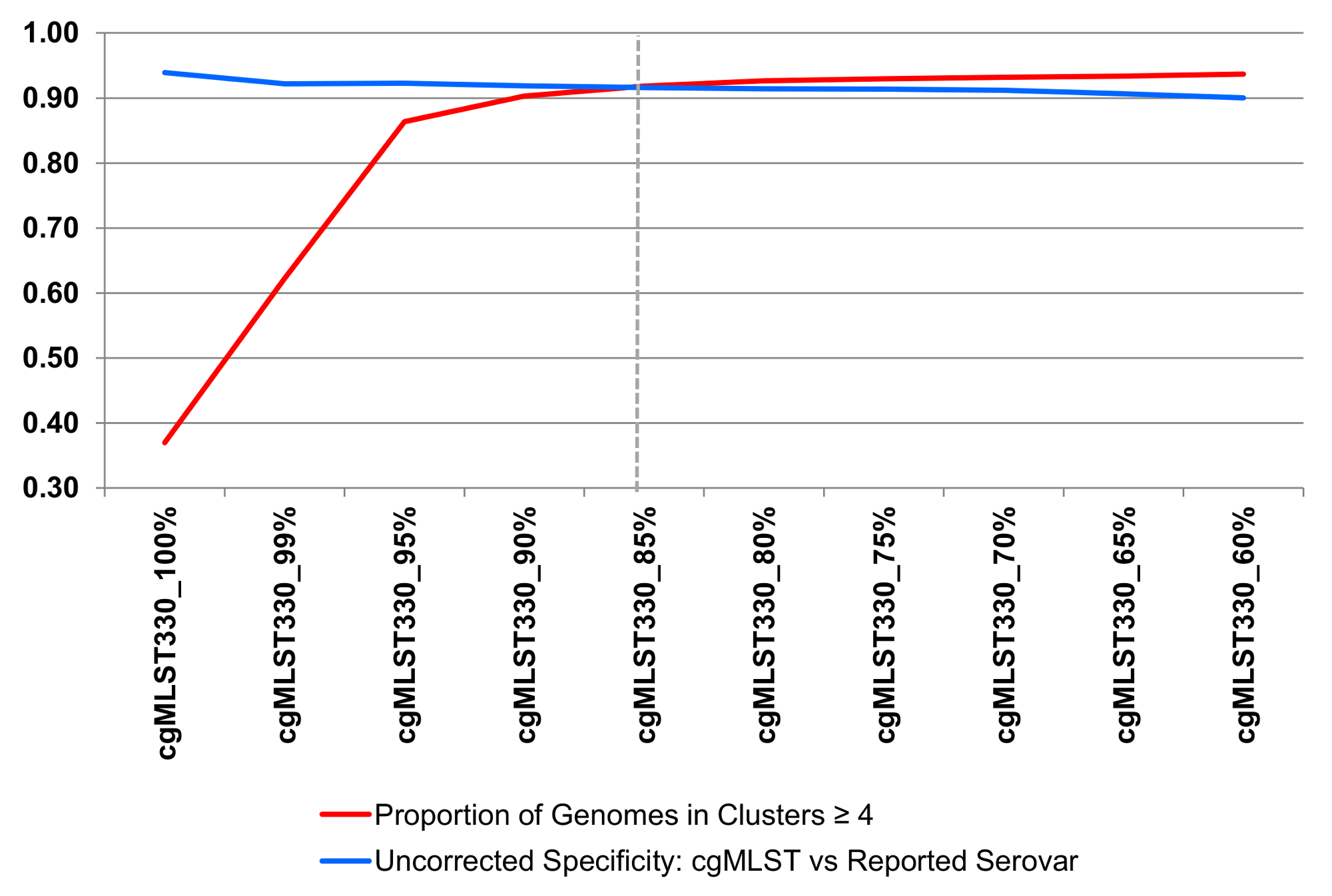

Supplement: S1 Fig — Analyses in this study were performed at a cgMLST clustering threshold of 85% profile similarity. This value maximized the proportion of genomes in clusters with a minimum cluster size of four without adversely affecting the specificity between cgMLST clusters and reported serovar. Cluster size was an important consideration in the analysis since, among cases where reported and predicted serovar did not match, genomes in cgMLST clusters smaller than four members (51 of 96 genomes with errors of Type 7) were not considered for cgMLST correction. (TIFF) [file pone.0147101.s001.tiff]
